# Psychometric properties and reference values of nine PROMIS parent-proxy measures for children aged 5 to 7 years in the Netherlands

**DOI:** 10.1186/s41687-026-01027-y

**Published:** 2026-03-02

**Authors:** Anne Westerweel, Michiel A. J. Luijten, Martha A. Grootenhuis, Caroline B. Terwee, Kelly L. A. van Bindsbergen, Lotte Haverman

**Affiliations:** 1 Princess Máxima Center for Pediatric Oncology, Utrecht, The Netherlands; 2 Amsterdam UMC Location University of Amsterdam, Emma Children’s Hospital, Child and Adolescent Psychiatry & Psychosocial Care, Amsterdam, The Netherlands; 3 Amsterdam Public Health, Mental Health, Amsterdam, The Netherlands; 4 Amsterdam Reproduction and Development, Child Development, Amsterdam, The Netherlands; 5 Amsterdam Public Health Research Institute, Methodology, Amsterdam, The Netherlands; 6 Department of Epidemiology and Data Science, Amsterdam UMC, Vrije Universiteit, Amsterdam, The Netherlands; 7 Amsterdam Public Health, Digital Health, Amsterdam, The Netherlands

**Keywords:** Patient-reported outcomes measurement information system (PROMIS®), Patient-reported outcome measures (PROMs), Parent-proxy reports, Psychometrics, Validity, Reliability, Computerized adaptive testing (CAT), Reference values, Dutch general population

## Abstract

**Background:**

The Patient-Reported Outcomes Measurement Information System (PROMIS®) provides standardized, generic measures to assess self-reported aspects of physical, mental, and social health. While PROMIS parent-proxy measures exist in multiple languages, evaluations outside the U.S. are limited. This study evaluated psychometric properties and provided Dutch reference values for PROMIS parent-proxy measures for children aged 5–7 years.

**Methodology:**

Parents (*n* = 529) completed PROMIS parent-proxy measures (Global Health, Anxiety, Depressive Symptoms, Anger, Peer Relationships, Mobility, Fatigue, Sleep Disturbance, Pain Intensity) and Pediatric Quality of Life Inventory (PedsQL) questionnaires as legacy measures. Structural validity was assessed using a Graded Response Model (GRM) with item fit statistics (S-X^2^, *p* < 0.001:misfit). Construct validity was evaluated by comparing PROMIS T-scores with PedsQL subscales. Reliability was estimated based on participants with a standard error (SE) ≤0.32 (reliability ≥0.90). Additionally, Test Information Functions (TIFs), and theoretical and empirical marginal reliability coefficients (>0.70:acceptable) were examined. Relative efficiency ((1-SE(*θ*)^2^)/n_items_) compared performance across PROMIS full form, short form, CAT, and PedsQL measures. Measurement invariance was assessed through Differential Item Functioning (DIF) analyses (McFadden’s pseudo *R*^2^ > 0.02:DIF), with impact evaluated by re-estimating GRMs without DIF item(s) and comparing T-scores, reliability indices, and Test Characteristic Curves (TCCs). Dutch reference values (mean T-score (*M*), standard deviation (*SD*)) were reported for PROMIS v1.0/3.0 and v2.0.

**Results:**

All PROMIS measures demonstrated acceptable psychometric properties. Five items showed misfit (Global Health:1; Fatigue:1; Sleep Disturbance:3), and three items exhibited DIF (Depressive Symptoms:1; Mobility:2), all with negligible impact. Construct validity confirmed alignment between PROMIS and PedsQL subscales. Reliability was high near the sample mean and extended into clinically relevant ranges. TIFs and marginal reliability coefficients confirmed good/excellent precision (0.85–0.94) for all item banks except for Mobility (0.56), with theoretical and empirical coefficients closely aligned. PROMIS CAT was most efficient, providing more information per item than the full form, short form, and PedsQL. Dutch reference values were obtained for v1.0/3.0 (*M*:[44.8–54.1], *SD*:[7.4–11.8]) and v2.0 (*M*:[44.5–50.7], *SD*:[9.1–12.5]).

**Conclusions:**

PROMIS parent-proxy Global Health, Anxiety, Depressive Symptoms, Anger, Peer Relationships, Mobility, Fatigue, Sleep Disturbance, and Pain Intensity measures showed acceptable psychometric properties in Dutch children aged 5–7 years. This study provides reference values for this population.

**Supplementary Information:**

The online version contains supplementary material available at 10.1186/s41687-026-01027-y.

## Background

Patient-Reported Outcome Measures (PROMs) are widely used in clinical care and research to assess Patient-Reported Outcomes (PROs) as perceived by the patient [1–5]. Recognizing the need for PROM standardization to enhance methodological rigor in research, improve data comparability in quality registries, and better inform clinicians, the Patient-Reported Outcomes Measurement Information System (PROMIS®) initiative was launched in 2002 [6, 7]. Investigators have since developed standardized PROMIS measures across various age groups: 1–4 years, 5–7 years, 8–17 years, and ≥18 years. These PROMIS measures are designed to be generic, ensuring their relevance and applicability across (disease) populations [6, 7]. Each PROMIS measure comprises a set of items designed to measure a singular construct, divided into three categories; physical, mental, and social health, related to emotions, symptoms or abilities (e.g., anger or mobility). Most PROMIS measures encompass an item bank that can be used as a concise short form (i.e., comprising a selection of specific items) or as a Computerized Adaptive Test (CAT; selecting items based on an individual’s preceding responses). The development of these measures was facilitated through the application of Item-Response Theory (IRT), where properties such as the difficulty and discriminative ability of each individual item are taken into consideration when computing the individual’s level within the respective construct [8]. By utilizing IRT modeling, items are systematically ordered based on their level of difficulty and discriminatory potential [9, 10]. This ordering process is essential for implementing CATs. Using CATs has several advantages, such as a heightened level of personalization and efficiency (e.g., fewer items need to be completed) within the PROM [11–13]. The scores of all measures developed from the same item bank (full item bank, short form, and CAT) are placed on the same scale, making them comparable.

PROMIS measures were originally developed and tested in the U.S. general population and in diverse patient populations [7, 14, 15]. To implement PROMIS in the Netherlands, the Dutch-Flemish PROMIS National Center (PNC) collaborated with other researchers to translate, gather reference values, and psychometrically evaluate several PROMIS measures for the Dutch-Flemish population [16–27]. However, the collection of reference values and the psychometric evaluation of PROMIS parent-proxy measures for children aged 5-7 years have not yet been investigated.

Therefore, the aim of this study was to assess the psychometric properties of PROMIS parent-proxy Anxiety (v3.0), Depressive Symptoms (v3.0), Peer Relationships (v3.0), Mobility (v3.0), Fatigue (v3.0), and Sleep Disturbance (v1.0) item banks, along with the Global Health scale (v3.0), Anger short form 5a (v3.0), and Pain Intensity item (v1.0) in a sample comprising parents with a child aged 5-7 years, living in the Netherlands. Additionally, this study aimed to gather Dutch reference values for these PROMIS measures.

## Methods

### Participants

Data were collected from parents with a child aged 5-7 years, living in the Netherlands, and having a sufficient understanding of the Dutch language to complete questionnaires. Parents were recruited through Flycatcher, an ISO-certified Dutch internet provider with a pre-profiled panel of respondents, between February and May 2024 (http://www.flycatcherpanel.nl). To assess the psychometric properties of PROMIS measures with a Graded Response Model (GRM), a sample size of 500 parents was considered adequate [28]. To ensure close alignment with the Dutch population for gathering reference data, we permitted a maximum deviation of 2.5% in key demographic characteristics compared to the Dutch general population, based on data from Statistics Netherlands (2023) (http://www.cbs.nl). These demographic characteristics encompassed the mean age and sex distribution of both children and parents, along with the child’s ethnicity (Dutch, Western immigrants, or non-Western immigrants), and parent’s educational level (low, middle, or high).

### Procedure

A two-step stratified sampling approach was employed to ensure a comprehensive representation of demographic characteristics for both children and parents. During the initial phase, participants were recruited from underrepresented strata based on the age and sex of both children and parents, the child’s ethnicity, and the parent’s educational level. Once the required number of participants was reached, or all potential participants from the underrepresented groups were contacted, the study proceeded to the second phase, inviting participants from remaining demographic strata until a minimum of 500 participants was reached.

Emails with information about the study and a link to the questionnaires were sent to parents with a child aged 5-7 years. Only one parent per household was invited, and only one child per family could be reported on. After reading the information form and signing online informed consent, participants completed the questionnaires of approximately 170 items. The survey was programmed with forced completion to avoid missing data; therefore, no imputation procedures were required. The study was exempt from the Medical Research Involving Human Subjects Act, as determined by the Medical Ethics Review Committee NedMec (number 24–002/DB).

### Measures

#### Demographic questionnaire

The demographic questionnaire contained questions about various aspects of the participant’s household. It included questions about the participating parent (sex, age, number of children, number of children living at home, marital status, work situation, ethnicity, educational level), the other parent (ethnicity), the participant’s current partner (work situation, educational level), and the child (sex, age, type of education, presence of a chronic physical, psychological, or intellectual condition).

#### PROMIS parent-proxy measures

Measures used for this study included Dutch-Flemish PROMIS parent-proxy Anxiety (v3.0), Depressive Symptoms (v3.0), Peer Relationships (v3.0), Mobility (v3.0), Fatigue (v3.0), and Sleep Disturbance (v1.0) item banks, along with the PROMIS parent-proxy Global Health scale (v3.0), Anger short form 5a (v3.0), and Pain Intensity item (v1.0). PROMIS item parameters were originally estimated using combined samples of the U.S. general population and children with chronic health conditions (v1.0 and v2.0) [7, 14, 15]. Recently, HealthMeasures released v3.0 of PROMIS pediatric and parent-proxy measures, in which item parameters were estimated based solely on the U.S. general population [29]. Additionally, some items were removed and response categories were collapsed compared to previous versions (Appendix A, Table A1) [29]. Consequently, v3.0 T-scores are not directly comparable to those of earlier versions, as they are based on different item parameters. To ensure alignment with the latest U.S.-based metrics, v3.0 measures were used in this study whenever available. Responses on short forms were derived from corresponding item banks: Anxiety 8a (8 items), Depressive Symptoms 6a (6 items), Peer Relationships 7a (7 items), Mobility 7a (7 items), Fatigue 10a (10 items), and Sleep Disturbance 8a (8 items).

PROMIS measures are answered on a five-point scale, typically ranging from 1 (*“Never”*) to 5 (*“Always”*), with a recall period of seven days. The PROMIS Pain Intensity item was rated on a 0 (“*No* *pain*”) to 10 (“*Worst pain you can think of*”) rating scale. PROMIS measures are scored on a T-score metric, with higher scores indicating higher levels of the specific assessed construct (i.e., higher levels of anxiety, better physical functioning, or more sleep disturbance) [10]. PROMIS measures were administered in their Dutch-Flemish psychometrically evaluated versions [16–27]. Further details on calibration and scoring of PROMIS measures, including the use of U.S. versus Dutch parameter sets, are described in the *Analyses* section.

#### Pediatric Quality of Life Inventory (PedsQL)

Two psychometrically evaluated Dutch parent-proxy modules (children aged 5–7 years) of the PedsQL were used as legacy instruments; the PedsQL Generic Core Scales (GCS) v4.0 [30–32], and the PedsQL Multidimensional Fatigue Scale (MFS) v3.0 [33, 34]. The PedsQL GCS assesses four domains: Physical (8 items), Emotional (5 items), Social (5 items), and School (5 items) functioning. The PedsQL MFS measures fatigue within cognitive (6 items), physical (6 items), and sleep/rest (6 items) dimensions. PedsQL measures are answered on a five-point scale, ranging from 1 (*“Never a problem”*) to 5 (*“Almost always a problem”*), with a recall period of seven days (acute version). Items are reverse-scored and linearly transformed to a 0–100 scale, with higher scores indicating better functioning (i.e., better physical functioning, better social functioning).

### Analyses

Analyses described below were performed for each PROMIS measure (Global Health, Anxiety, Depressive Symptoms, Anger, Peer Relationships, Mobility, Fatigue, Sleep Disturbance). Since PROMIS Pain Intensity consists of a single item, only construct validity was investigated. Psychometric properties of scores were assessed using the total study sample, while Dutch reference values were calculated from the reference sample.

#### PROMIS scoring and calibration

Two sets of item parameters were used in this study. To derive Dutch reference values and to evaluate construct validity, T-scores were calculated using the official PROMIS parent-proxy v1.0, v2.0 and v3.0 item parameters implemented in the HealthMeasures Scoring Service (http://www.healthmeasures.net) [10]. These parameters are not publicly released in scoring manuals or publications, but represent the official and internationally applied parameters for PROMIS scoring. A T-score of 50 (*SD* = 10) reflects the mean of the HealthMeasures reference population [35]. For all other psychometric evaluations within the Dutch study sample, including structural validity, measurement invariance, reliability, and relative efficiency, item parameters were estimated from the Dutch data. To enable direct comparison of efficiency ratios between measures, item parameters were also estimated for the PedsQL GCS and MFS (sub)scales using GRM on the Dutch data.

#### Psychometric properties

##### Structural validity

Following the PROMIS analysis plan for psychometric evaluation of PROMIS measures, structural validity was first evaluated by checking underlying assumptions of unidimensionality, local independence, and monotonicity. Unidimensionality was assessed through confirmatory factor analysis (CFA) and exploratory bi-factor analysis. CFAs were conducted using the WLSMV estimator with a probit link on polychoric correlations using R package ‘lavaan’ (version 0.6–17) [36]. The latent variable variance was fixed to 1 for model identification. Model fit was evaluated with the Comparative Fit Index (CFI), Tucker-Lewis Index (TLI), Root Mean Square Error of Approximation (RMSEA), and Standardized Root Mean Residual (SRMR), with CFI/TLI ≥0.95, RMSEA ≤0.06, and SRMR ≤0.08 indicating good fit. Standardized factor loadings were inspected for all items. Exploratory bi-factor analyses were used to further evaluate essential unidimensionality, without interpreting domain-specific factors, with Explained Common Variance (ECV ≥0.70) and Omega-H (ωh ≥0.80) considered supportive. Local independence was assessed by examining residual correlations from the CFA model, with values >0.20 indicating potential dependence. Monotonicity was evaluated with Mokken scale analysis using R package ‘Mokken’ (version 3.2.1), with scalability coefficients H_i_ ≥0.30 (item level) and H ≥0.50 (scale level) considered acceptable [37, 38]. Invariant item ordering (IIO) was additionally examined to describe the extent to which items can be ordered similarly across participants, with HT coefficients <0.30 indicating inaccurate ordering, 0.30–0.39 low accuracy, 0.40–0.49 medium accuracy, and ≥0.50 high accuracy [39]. Since IIO is not a required assumption for GRMs, HT results were not used to evaluate structural validity.

Once the assumptions were supported, a GRM was fitted to estimate discrimination (α) and difficulty (β) parameters using R package ‘mirt’ (version 1.41.0) [40]. Because ordinal CFA and GRM are mathematically related, CFA was first applied to evaluate unidimensionality, after which the GRM was used to estimate item parameters and item fit. Item fit within the GRM was investigated using generalized Orlando and Thissen’s S-X^2^ for polytomous data [41], with *p* <0.001 indicating item misfit [42]. When an item showed misfit, item information and item-fit plots were visually inspected to evaluate its impact. Misfit was considered negligible if the item provided sufficient information across the relevant theta range with logically ordered response options (item information plot), and if observed and expected responses showed minor deviations across the theta range (item-fit plots).

##### Construct validity

For the evaluation of construct validity of scores, U.S.-based T-scores for each PROMIS measure were compared with corresponding PedsQL (sub)scale scores. Based on literature, strong correlations (Pearson’s ∣*r*∣ >0.50) were expected between PROMIS Anger, Anxiety, and Depressive Symptoms and the PedsQL GCS emotional subscale [27, 43, 44], between PROMIS Mobility and the PedsQL GCS physical subscale [43, 44], and between PROMIS Fatigue and the PedsQL MFS total score [43–45]. Moderate correlations (Pearson’s ∣*r*∣ >0.30) were expected between PROMIS Global Health and the PedsQL GCS total score [46, 47], and between PROMIS Peer Relationships and the PedsQL GCS social subscale [21, 43, 44]. No hypotheses based on correlations found in prior studies could be formulated for PROMIS Sleep Disturbance and Pain Intensity measures. However, we hypothesized a strong correlation (Pearson’s ∣*r*∣ >0.50) between the PROMIS Sleep Disturbance measure and the PedsQL MFS sleep subscale, and between the PROMIS Pain Intensity item and item 7 (*“having pain”*) of the PedsQL GCS.

##### Reliability

PROMIS measures are scored using IRT, where each response pattern is associated with a standard error (SE) of the estimated construct (*θ*) [10]. The standard error of theta (SE(*θ*)) reflects score reliability, with values ≤0.32 corresponding to reliability of ≥0.90, a commonly accepted threshold for high reliability in IRT-based measures. Reliability of scores from PROMIS item banks and extracted short forms was estimated by determining the number of participants whose θ had a corresponding SE(*θ*) ≤0.32, calculated through the Expected A Posteriori (EAP) estimator.

Reliability of scores from PROMIS CATs was assessed with a post hoc CAT simulation using the Maximum Posterior Weighted Information (MPWI) selection criterion and EAP estimator [48] with R package ‘catR’ (version 3.17) [49]. Standard start and stopping PROMIS CAT rules were used for the post hoc CAT simulation [50]. The CAT started with the item providing the most information at the mean of the construct (*θ*=0), after which subsequent items were selected to maximize MPWI at the current EAP *θ*-estimate. The CAT was stopped either when the SE(*θ*) ≤0.32 was achieved or when a minimum of four items and a maximum of × items was reached (where × equals the length of the specific short form). To assess reliability of scores on corresponding PedsQL subscales, a GRM was fitted on each subscale of the PedsQL. Using the same approach as for PROMIS measures, score reliability was estimated by calculating the number of participants whose *θ* had a corresponding SE(*θ*) ≤0.32, using the EAP estimator.

To account for potential limitations at scale extremes, reliability was assessed in both the total study sample and a subgroup excluding participants with extreme raw summed scores on PROMIS measures (i.e., those with the best possible functioning). In addition, Test Information Functions (TIFs) were computed for each PROMIS full item bank to evaluate measurement precision across the *θ*-scale. Higher information values indicated greater measurement precision (i.e., more reliable scores). From the TIFs, two marginal reliability coefficients were calculated using R package ‘mirt’ (version 1.41.0) [40]. The theoretical marginal reliability coefficient reflects measurement precision under the assumption of a standard normal *θ*-distribution, whereas the empirical marginal reliability coefficient reflects measurement precision given the observed *θ*-distribution in the study sample. Both indices range from 0 to 1, with values of 0.70–0.80, 0.81–0.90, and >0.90 considered acceptable, good, and excellent respectively.

Additionally, efficiency was computed across different measures, including the item bank, extracted short form, simulated CAT, and corresponding PedsQL (sub)scale, for each participant by dividing the total test information by the number of items, calculated as ((1- SE(*θ*)^2^)/n_items_). Relative efficiency was then calculated as the ratio of mean efficiencies between these measures. To quantify uncertainty, 95% bootstrap confidence intervals (1,000 resamples) were calculated for the efficiency ratios. Additionally, observed *θ*-ranges across participants were determined for each measure to indicate sample dependence of the results. The PedsQL is normally scored using classical test theory (0–100 transformed scores). However, a GRM was fitted to the PedsQL (sub)scales to estimate SEs and test information. All item parameters (PROMIS and PedsQL) were estimated from the Dutch data, to ensure direct comparability of efficiency estimates.

##### Measurement invariance (Differential item functioning (DIF) analysis)

Measurement invariance was assessed for age, sex, and ethnicity of children, and age, sex, and educational level of parents using DIF analysis. Both uniform and non-uniform DIF were investigated between groups by calculating McFadden’s pseudo *R*^2^ using R package ‘lordif’ (version 0.3–3) [51]. McFadden’s pseudo *R*^2^ >0.02 was indicative of DIF. For group comparisons involving more than two categories, such as educational level (low, middle, high), the mean McFadden’s pseudo *R*^2^ was reported, while individual pairwise comparisons were also checked for DIF.

When an item was flagged for DIF, its impact was examined by comparing T-scores and reliability estimates between the original application forms (full item bank, short form, and CAT) and versions with the DIF item(s) removed. For each application, a GRM was re-estimated without the DIF item(s), and the resulting T-scores and reliability estimates (mean SE(*θ*) and % of participants with SE(*θ*) ≤0.32) were compared with those of the original form. The mean absolute and maximum T-score differences, as well as the proportion of participants with a difference ≥1 T-score were reported. Additionally, Test Characteristic Curves (TCCs) were visually inspected, and the impact of DIF was considered negligible when curves showed minor deviations in the *θ*-range where participants were located.

#### Dutch reference values

To derive Dutch reference values, mean (standard deviation (*SD*)) T-scores were determined for each currently recommended PROMIS measure (Global Health (v3.0), Anxiety (v3.0), Depressive Symptoms (v3.0), Anger (v3.0), Peer Relationships (v3.0), Mobility (v3.0), Fatigue (v3.0), Sleep Disturbance (v1.0)). Additionally, Dutch reference values were established for previous v2.0 of PROMIS Anxiety, Depressive Symptoms, Anger, Peer Relationships, Mobility, and Fatigue measures, as these are still in use and no Dutch reference values were available. Responses to v2.0 items were extracted from collected PROMIS v3.0 data in this study sample. Consequently, one item of PROMIS Peer Relationships and three items of Mobility measures were not included in the T-score calculation. Therefore, the v2.0 reference values should be considered provisional, as they were derived from v3.0 measures in which not all v2.0 items were included. SE(*θ*) values were compared between PROMIS v2.0 and v3.0 for each measure, to evaluate measurement reliability and potential CAT efficiency. T-scores were calculated for the entire reference sample, as well as for male and female children and parents separately.

## Results

### Participants

In total, 370 respondents were included in the initial phase to form the reference sample, which was closely aligned with the Dutch general population on key demographic characteristics. During the second phase, 159 additional participants were recruited from the remaining demographic strata to reach the required minimum of 500 respondents for psychometric analyses. In total, 4068 parents were invited to participate in the study, and 529 parents completed the questionnaires (response rate: 13.0%). Table 1 presents the demographic characteristics of both the reference and the total study samples. Female parents were overrepresented in the reference sample by more than 2.5% compared to the Dutch general population.

**Table 1:** Participant characteristics of both reference and total study samples

Demographic characteristic	Reference sample (*n* = 370)	Total study sample (*n* = 529)	Dutch population in 2023^a^
**Child**			
**Age in years [mean ± SD]**	6.0 ± 0.8	6.0 ± 0.8	6.0 ± NA
**Sex (female)**	48.6	49.0	48.7
**Ethnicity**			
Dutch	65.4	71.6	65.0
Western immigrant	9.7	8.9	10.0
Non-Western immigrant	24.9	18.7	25.0
Unknown	0.0	0.8	
**Education level (regular primary)**	97.6	97.4	
**Presence of chronic condition**	13.2	13.2	
**Parent: Participant**			
**Age in years [mean ± SD]**	38.4 ± 5.2	38.3 ± 5.2	39.3 ± NA
**Sex (female)**	65.7	77.2	56.0
**Ethnicity**			
Dutch	77.6	81.9	
Western immigrant	9.2	10.0	
Non-Western immigrant	13.2	7.6	
Unknown	0.0	0.6	
**Education level**^**b**^			
Low	12.7	11.0	13.5
Medium	36.2	33.6	36.0
High	51.1	55.4	50.5
**Work situation**			
Employed	85.1	86.8	
Unemployed/Job seeking	8.9	7.9	
Studying	0.3	0.6	
Unfit for work	5.7	4.7	
**Marital status**			
Married/Cohabitating	85.1	86.6	
Divorced	6.5	5.9	
Widow/Widower	0.5	0.6	
Single	7.3	6.6	
Other	0.5	0.4	
**Number of children [mean ± SD]**	2.4 ± 1.0	2.4 ± 1.0	
**Number of children living home [mean ± SD]**	2.3 ± 1.0	2.3 ± 0.9	
**Other parent**			
**Ethnicity**			
Dutch	75.1	79.6	
Western immigrant	7.3	6.0	
Non-Western immigrant	17.6	13.6	
Unknown	0.0	0.8	
**Participant’s partner**			
**Education level**^**b**^			
Low	11.4	10.5	
Medium	37.8	36.5	
High	50.8	53.0	
**Work situation**			
Employed	90.2	91.5	
Unemployed/Job seeking	6.0	5.5	
Unfit for work	3.5	2.8	
Studying	0.3	0.2	

Values are expressed in percentages (%) unless stated otherwise. SD, standard deviation; NA, not applicable

^a^ Dutch population of children aged 5-7 years and their parents; used to ensure a study sample with a maximum deviation of 2.5% in key demographic characteristics. Based on data from Statistics Netherlands (http://www.cbs.nl)

^b^ Low: primary, lower vocational, lower general, and middle general education; middle: middle vocational, higher secondary, and pre-university education; high: bachelor’s degree, master’s degree, doctorate

### Structural validity

IRT-assumptions (Table 2) and model fit (Appendix B, Table B1) were checked for each PROMIS measure. CFA results indicated good fit for most indices, although unidimensionality was not supported by RMSEA (values >0.06) except for Mobility. Standardized factor loadings from the CFAs were moderate to high across all item banks (ranges 0.52–0.95). While CFA alone did not fully support unidimensionality, exploratory bi-factor analysis demonstrated that essential unidimensionality was met, with ωh values ≥0.80 and ECV values ≥0.70 for all measures. Local independence checks indicated one flagged item pair within the Global Health scale (*PedGlobal5_PXR1* and *PedGlobal6_PXR1*; residual correlation = 0.271; 3.6% of item pairs). No other item pairs showed local dependence, and all measures demonstrated adequate monotonicity (H_i_ ≥0.30, H ≥0.60). IIO analyses showed low ordering accuracy across most domains (HT = 0.04–0.36), except for Anger and Mobility (HT = 0.49–0.54). Together, these findings indicate that the assumptions of unidimensionality, local independence, and monotonicity were met across all PROMIS measures.

**Table 2:** Results of the IRT-assumptions for each PROMIS parent-proxy measure (*n* = 529)

Assumption - Analysis	Outcome	PROMIS parent-proxy measure							
		Global Health	Anxiety	Depressive Symptoms	Anger	Peer Relationships	Mobility	Fatigue	Sleep Disturbance
Unidimensionality - Confirmatory Factor Analysis	Scaled CFI	0.974	0.986	0.993	0.993	0.984	0.999	0.994	0.957
	Scaled TLI	0.961	0.983	0.991	0.986	0.981	0.999	0.993	0.950
	Scaled RMSEA	0.228*	0.093*	0.079*	0.122*	0.097*	0.031	0.066*	0.140*
	SRMR	0.081*	0.044	0.033	0.029	0.029	0.024	0.028	0.074
	Standardized factor loading (range)	0.523–0.949	0.730–0.894	0.757- 0.936	0.801–0.904	0.780- 0.899	0.516–0.988	0.772–0.949	0.529- 0.952
Unidimensionality - Exploratory bi-factor analysis	ECV	0.712	0.792	0.903	0.855	0.926		0.905	0.763
	Omega-H (ωh)	0.801	0.842	0.912	0.886	0.964		0.932	0.864
Local dependence	Residual correlation >0.2 (number (percentage) of locally dependent items)	1 (3.6)*	0 (0.0)	0 (0.0)	0 (0.0)	0 (0.0)	0 (0.0)	0 (0.0)	0 (0.0)
Monotonicity	Scalability coefficient H	0.578	0.668	0.705	0.711	0.689	0.742	0.738	0.549
	Scalability coefficient H_i_ (range)	0.447–0.663	0.583–0.711	0.607- 0.747	0.687–0.735	0.625- 0.726	0.413–0.823	0.622–0.782	0.318- 0.653
	Invariant item ordering HT	0.036	0.356	0.282	0.495	0.120	0.541	0.187	0.214

CFI, comparative fit index; TLI, Tucker-Lewis index; RMSEA, root mean square error of approximation; SRMR, standardized root mean residual; ECV, explained common variance

*Outcome does not satisfy the required IRT-assumption (for unidimensionality, local dependence, and monotonicity). For invariant item ordering, values are reported descriptively, as it is not a required assumption for graded response models

One item from the PROMIS Global Health scale (*PedGlobal2_PXR1*), one from the PROMIS Fatigue measure (*Pf4fatigue11r*), and three from the PROMIS Sleep Disturbance measure (*sq007p_r, sq020p_r, and sq023p*) showed item misfit (S-X^2^* p-*value <0.001). However, item information plots showed that these items still provided adequate coverage across the relevant *θ*-range with logically ordered response options (Appendix C, Figure C1, first column), and the item-fit plots showed only minor deviations between observed and expected responses (Appendix C, Figure C1, second column). Therefore, the impact of item misfit was considered negligible.

### Construct validity

T-scores of PROMIS Anxiety and Depressive Symptoms measures showed strong correlations with the PedsQL emotional subscale (∣*r*∣ >0.70). PROMIS Anger, Fatigue, Sleep Disturbance, and Pain Intensity measures showed moderately strong correlations (∣*r*∣ >0.50), while PROMIS Global Health, Peer Relationships, and Mobility measures showed moderate correlations (∣*r*∣ >0.30) with corresponding PedsQL (sub)scales. Whereas most results were consistent with expectations (Table 3), the PROMIS Mobility measure showed a moderately strong correlation with the PedsQL physical subscale (∣*r*∣ =0.658), which was lower than the hypothesized strong correlation (∣*r*∣ >0.70).

**Table 3:** Pearson correlation coefficients between each PROMIS measure and corresponding PedsQL (sub)scale (*n* = 529)

PROMIS parent-proxy measure	Corresponding PedsQL (sub)scale	Expected Pearson correlation (∣*r*∣)	Observed Pearson correlation (∣*r*∣)
Global Health	Total sum score GCS	>0.30	0.448
Anxiety	Emotional scale GCS	>0.70	0.759
Depressive Symptoms	Emotional scale GCS	>0.70	0.760
Anger	Emotional scale GCS	>0.50	0.638
Peer Relationships	Social scale GCS	>0.30	0.364
Mobility	Physical scale GCS	>0.70	0.658*
Fatigue	Total sum score MFS	>0.50	0.708
Sleep Disturbance	Sleep/Rest scale MFS	>0.50	0.652
Pain Intensity	Item 7 GCS	>0.50	0.513

GCS, general core scales; MFS, multidimensional fatigue scale

*Expected and observed Pearson correlations do not align

### Reliability

**Fig. 1:**
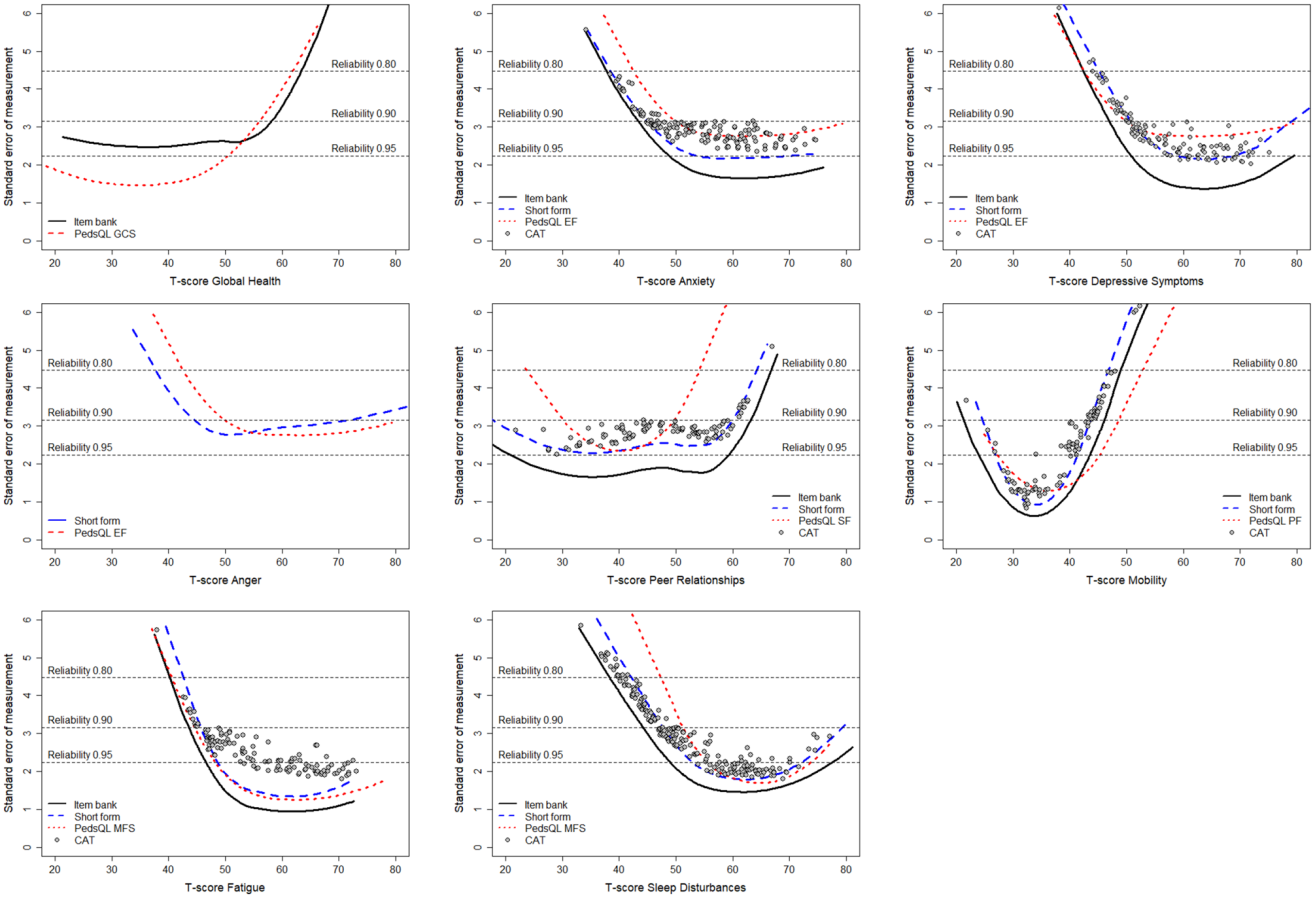
Standard errors of measurements across T-scores for PROMIS and PedsQL measures, using Dutch parameters (*n* = 529). GCS, generic core scales; EF, emotional functioning; SF, social functioning; PF, physical functioning; MFS, multidimensional fatigue scales

Scores from PROMIS Global Health, Anxiety, Depressive Symptoms, Anger, Peer Relationships, Fatigue, and Sleep Disturbance measures demonstrated reliability ≥0.90 at the sample mean (T-score = 50) and extended ≥2SDs into the clinically relevant ranges, based on Dutch item parameters (Fig. 1). PROMIS Mobility scores showed lower reliability, with 0.75 at the sample mean, but exceeded 0.90 within the T-score range of 25–45.

Based on SE(*θ*) ≤0.32, the majority of T-scores were reliable for PROMIS Global Health (75%), Anxiety (>70%), Anger (66%), Peer Relationships (>80%), Fatigue (>63%), and Sleep Disturbance (>58%) across all applications (item bank, short form, and CAT) (Table 4). In contrast, the percentage of reliable T-scores was lower for Depressive Symptoms (>39%) and Mobility (>18%) measures. However, after excluding extreme raw summed scores (i.e., parents reporting the best possible functioning in their child), reliability notably improved, particularly for Depressive Symptoms, Mobility, and Fatigue measures.

**Table 4:** Score reliability of measurements for PROMIS item bank, short form, CAT, and corresponding PedsQL (sub)scale

	PROMIS item bank			PROMIS CAT			PROMIS short form			PedsQL (sub)scale		
	Mean SE(*θ*)	% SE(*θ*) ≤0.32*	No. of items	Mean SE(*θ*)	% SE(*θ*) ≤0.32*	No. of items	Mean SE(*θ*)	% SE(*θ*) ≤0.32*	No. of items	Mean SE(*θ*)	% SE(*θ*) ≤0.32*	No. of items
**Global Health**												
Total Sample^a^	0.303	74.7	7							0.268	75.0	23
Without ceiling effect^b^ (*n* = 520)	0.297	76.0	7							0.266	75.4	23
**Anxiety**												
Total Sample^a^	0.267	75.2	13	0.331	71.3	5.7	0.304	70.1	8	0.376	44.6	5
Without ceiling effect^b^ (*n* = 469)	0.231	84.9	13	0.302	80.4	5.5	0.271	79.1	8	0.358	49.7	5
**Depressive Symptoms**												
Total Sample^a^	0.316	57.7	13	0.378	44.8	5.3	0.394	39.7	6	0.376	44.6	5
Without ceiling effect^b^ (*n* = 425)	0.247	71.8	13	0.321	55.8	5.1	0.336	49.4	6	0.351	52.9	5
**Anger**												
Total Sample^a^							0.330	66.2	5	0.376	44.6	5
Without ceiling effect^b^ (*n* = 481)							0.308	72.8	5	0.363	48.6	5
**Peer Relationships**												
Total Sample^a^	0.221	86.8	14	0.318	82.4	4.8	0.287	80.9	7	0.424	43.9	5
Without ceiling effect^b^ (*n* = 476)	0.191	96.4	14	0.296	91.6	4.5	0.262	89.9	7	0.412	47.1	5
**Mobility**												
Total Sample^a^	0.498	28.9	20	0.547	20.0	6.5	0.613	18.1	7	0.406	38.9	8
Without ceiling effect^b^ (*n* = 333)	0.361	45.9	20	0.431	31.8	6.1	0.530	28.8	7	0.348	52.3	8
**Fatigue**												
Total Sample^a^	0.251	70.7	23	0.334	64.3	6.4	0.319	63.3	10	0.277	68.6	18
Without ceiling effect^b^ (*n* = 408)	0.160	91.7	23	0.264	83.3	5.4	0.241	82.1	10	0.237	78.2	18
**Sleep Disturbance**												
Total Sample^a^	0.266	71.3	15	0.318	62.6	5.8	0.326	58.0	8	0.425	38.0	6
Without ceiling effect^b^ (*n* = 492)	0.243	76.6	15	0.298	67.3	5.7	0.305	62.4	8	0.328	58.0	6

CAT, computerized adaptive testing; SE, standard error of measurement; *θ*, theta

^a^ Total sample of participants (*n* = 529)

^b^ Sample of participants excluding those with extreme raw summed scores

*Percentage of participants with reliable measurements (SE(*θ*) ≤0.32)

In addition, TIFs and marginal reliability coefficients were calculated for each PROMIS full item bank (Appendix E, Table E1 and Figure E1). Empirical marginal reliability indicated good to excellent measurement precision for all measures (range: 0.85–0.94), except for PROMIS Mobility (0.56). Theoretical and empirical marginal reliability coefficients aligned for all measures (difference range: 0.00–0.02), except for PROMIS Mobility (difference: 0.06), suggesting that measurement precision under standard IRT-assumptions closely aligned with measurement precision in our study sample.

When comparing PROMIS measures to corresponding PedsQL (sub)scales, the percentage of reliable T-scores was at least 20% higher for PROMIS Anxiety, Anger, Peer Relationships, and Sleep Disturbance measures. PROMIS Global Health, Depressive Symptoms, and Fatigue measures showed similar reliability to their corresponding PedsQL (sub)scales (mean difference $\approx$ 3%). In contrast, PROMIS Mobility showed lower reliability than the corresponding PedsQL subscale (difference $\approx$ 17%). Across all measures, CAT was most efficient, providing more information per item than the full item bank, short form, and PedsQL (sub)scales (Table 5). Bootstrap 95% confidence intervals and observed *θ*-ranges are reported to illustrate uncertainty and sample dependence of the efficiency ratios (Table 5).

**Table 5:** Relative efficiency ratios (bootstrap 95% confidence intervals) of PROMIS item bank, short form, CAT, and PedsQL (sub)scales with observed *θ*-ranges (*n* = 529)

		PROMIS item bank	PROMIS short form	PROMIS CAT	*θ*-range
Global Health	PedsQL GCS	1.76 [1.66–1.85]	-	-	−3.15–1.92
Anxiety	PedsQL EF subscale	0.98 [0.94–1.02]	1.08 [1.05–1.13]	2.85 [2.71–3.00]	−1.60–2.93
	PROMIS item bank	-	1.11 [1.09–1.12]	2.92 [2.85–2.98]	
	PROMIS short form	-	-	2.63 [2.56–2.70]	
Depressive Symptoms	PedsQL EF subscale	0.95 [0.90–1.00]	1.01 [0.96–1.06]	2.85 [2.67–3.03]	−1.27–3.36
	PROMIS item bank	-	1.06 [1.04–1.09]	3.00 [2.93–3.06]	
	PROMIS short form	-	-	2.82 [2.73–2.90]	
Anger	PedsQL EF subscale	-	1.19 [1.14–1.24]	-	−1.62–3.46
Peer Relationships	PedsQL SF subscale	1.06 [1.00–1.14]	1.09 [1.03–1.17]	3.47 [3.27–3.73]	−3.63–1.79
	PROMIS item bank	-	1.02 [1.01–1.04]	3.27 [3.20–3.32]	
	PROMIS short form	-	-	3.19 [3.11–3.26]	
Mobility	PedsQL PF subscale	0.81 [0.70–0.91]	0.99 [0.84–1.14]	3.53 [2.99–4.04]	−2.93–0.93
	PROMIS item bank	-	1.23 [1.16–1.29]	4.38 [4.11–4.64]	
	PROMIS short form	-	-	3.56 [3.30–3.87]	
Fatigue	PedsQL MFS	1.30 [1.23–1.37]	1.55 [1.46–1.64]	7.15 [6.71–7.62]	−1.30–2.84
	PROMIS item bank	-	1.19 [1.17–1.22]	5.52 [5.38–5.65]	
	PROMIS short form	-	-	4.62 [4.51–4.74]	
Sleep Disturbance	PedsQL MFS Sleep/Rest subscale	0.91 [0.85–0.98]	1.13 [1.06–1.22]	3.11 [2.89–3.34]	−1.70–3.12
	PROMIS item bank	-	1.24 [1.22–1.27]	3.42 [3.34–3.49]	
	PROMIS short form	-	-	2.74 [2.67–2.81]	

*θ*, theta; GCS, generic core scales; EF, emotional functioning; SF, social functioning; PF, physical functioning; MFS, multidimensional fatigue scales. A relative efficiency ratio exceeding 1.00 signifies that the column is more efficient than the row

### Measurement invariance (DIF analysis)

No DIF was found for PROMIS Global Health, Anxiety, Anger, Peer Relationships, Fatigue, and Sleep Disturbance measures (Appendix B, Table B1). Within the PROMIS Depressive Symptoms measure, one item (*Pf2depr1r2*) was flagged for DIF related to educational level (non-uniform DIF between low and high educational levels (*R*^2^ = 0.0267); uniform DIF between medium and high educational levels (*R*^2^ = 0.0232)). Removing this item from the full item bank had minimal impact on T-scores (mean absolute difference = 0.26; maximum difference = 4.7; % of participants ≥1 T-score difference = 3.6%) and reliability (mean SE(*θ*) increased from 0.316 to 0.321; % of participants with mean SE(*θ*) ≤0.32 decreased from 57.7% to 56.7%). TCCs confirmed negligible impact (Appendix D, Figure D1, first and second column). In the CAT simulation, this item was not administered to any participant. Item information peaked at *θ* =1.4, overlapping with 33% of participants, but it was never the most informative item at any participant’s *θ*-level and was included among the top three of most informative items only in 20% of participants. Therefore, its practical impact on T-scores and reliability in CATs was considered negligible.

In the PROMIS Mobility measure, two items were flagged for DIF related to the child’s age. Item *Pf3mobil8r2* demonstrated uniform DIF between children aged 5 and 6 years (*R*^2^ = 0.020) and non-uniform DIF between children aged 5 and 7 years (*R*^2^ = 0.0269). Item *Pf4mobil9r2* was flagged for uniform DIF between children aged 5 and 7 years (*R*^2^ = 0.0236). Removing this item from the full item bank had minimal impact on T-scores (mean absolute difference = 0.25; maximum difference = 8.1; % of participants ≥1 T-score difference = 4.4%) and reliability (mean SE(*θ*) increased from 0.498 to 0.501; % of participants with mean SE(*θ*) ≤0.32 decreased from 28.9% to 28.0%). TCCs confirmed negligible impact (Appendix D, Figure D1, third and fourth column). For the short form (which included DIF item *Pf3mobil8r2*), removing this item had minimal impact on T-scores (mean absolute difference = 0.13; maximum difference = 12.9; % of participants ≥1 T-score difference = 1.1%) and reliability (mean SE(*θ*) increased from 0.613 to 0.617; % of participants with mean SE(*θ*) ≤0.32 decreased from 18.1% to 17.8%). In the CAT simulation, none of the DIF items were administered to any participant. Item information peaked at *θ* =−1.5 to −1.8, overlapping with 11%-17% of participants. Therefore, their practical impact on T-scores and reliability in CATs was considered negligible.

Across all PROMIS measures, only three items were flagged for DIF. Effect-size analyses on T-score and reliability differences showed negligible impact across all applications, supporting cross-group comparability of the measures.

### Dutch reference values

Dutch reference values were obtained for each PROMIS measure (Table 6). For PROMIS v1.0/v3.0 measures, T-scores in the Dutch general population ranged from 44.8 to 54.1, with SDs between 7.4 and 11.8. The mean score for the PROMIS Pain Intensity item was 3.1 (*SD* = 2.5). For PROMIS v2.0 measures, Dutch T-scores ranged from 44.5 to 50.7, with SDs between 9.1 and 12.5. Although identical response patterns were used to calculate T-scores for v3.0 and v2.0, T-scores differed between those versions due to the application of distinct IRT models. Importantly, mean SE(*θ*) was lower for v3.0 than for v2.0, except for Mobility, indicating higher score precision. This suggests that CATs based on v3.0 may require fewer items to achieve reliable scores (Table 6).

**Table 6:** Reference values for PROMIS measures v1.0/v3.0 and v2.0 in the Dutch general population

	Administered version (v1.0/v3.0)					v2.0^**^				
	Total sample* (*n* = 370)	Sex child		Sex parent		Total sample* (*n* = 370)	Sex child		Sex parent	
		Boys (*n* = 190)	Girls (*n* = 180)	Male (*n* = 127)	Female (*n* = 243)		Boys (*n* = 190)	Girls (*n* = 180)	Male (*n* = 127)	Female (*n* = 243)
Global Health (v3.0)	46.7 (10.0)	46.3 (9.8)	47.1 (10.2)	45.8 (10.1)	47.2 (9.9)	-	-	-	-	-
Anxiety (v3.0)	53.3 (8.6) *Mean SE(θ)*=2.4	53.8 (9.0)	52.8 (8.2)	53.6 (9.3)	53.1 (8.2)	50.7 (10.7) *Mean SE(θ)*=3.1	51.3 (11.1)	50.0 (10.1)	51.3 (11.4)	50.4 (10.3)
Depressive Symptoms (v3.0)	50.7 (9.6) *Mean SE(θ)*=2.9	51.4 (10.1)	49.9 (9.1)	51.7 (10.6)	50.2 (9.1)	45.7 (11.3) *Mean SE(θ)*=3.6	46.6 (11.9)	44.7 (10.6)	47.1 (12.4)	44.9 (10.6)
Anger (v3.0)	54.1 (8.8)	55.4 (9.1)	52.7 (8.2)	53.8 (9.2)	54.2 (8.6)	-	-	-	-	-
Peer Relationships (v3.0)	50.1 (9.3) *Mean SE(θ)*=2.3	49.0 (9.2)	51.2 (9.2)	47.6 (10.1)	51.5 (8.5)	45.4 (9.1) *Mean SE(θ)*=2.5	44.3 (8.9)	46.6 (9.2)	43.2 (9.7)	46.6 (8.5)
Mobility (v3.0)	44.8 (11.8) *Mean SE(θ)*=5.8	42.9 (12.9)	46.8 (10.3)	42.4 (13.7)	46.1 (10.5)	50.1 (10.4) *Mean SE(θ)*=4.5	48.4 (11.4)	51.8 (8.9)	47.7 (12.3)	51.3 (9.0)
Fatigue (v3.0)	52.3 (10.6) *Mean SE(θ)*=2.7	53.2 (11.0)	51.4 (10.2)	54.0 (11.4)	51.4 (10.1)	44.5 (12.5) *Mean SE(θ)*=3.0	45.6 (12.9)	43.3 (11.8)	46.7 (13.4)	43.3 (11.8)
Sleep Disturbance (v1.0)	53.1 (9.8)	53.4 (10.3)	52.7 (9.3)	54.3 (10.1)	52.4 (9.6)	-	-	-	-	-

SD, standard deviation; SE, standard error of theta; *θ*, theta

*The U.S. T-score (*SD*) is 50.0 (10.0) for all PROMIS measures, reflecting the mean of the HealthMeasures reference population, not necessarily the mean of the U.S. general population

**Responses to v2.0 items were extracted from collected PROMIS v3.0 data in this study sample (Appendix A, Table A1). Consequently, one item of PROMIS Peer Relationships and three items of Mobility measures were not included in the T-score calculation. Therefore, v2.0 values should be interpreted as provisional reference values

Fathers generally rated their children as having poorer outcomes on PROMIS measures–except for Anger–compared to mothers. For example, fathers reported slightly higher pain levels in their children (*M* = 3.3, *SD* = 2.6) than mothers (*M* = 3.1, *SD* = 2.4). Regarding the sex of the child, parents reported lower T-scores for boys compared to girls across all measures. Additionally, pain levels were reported higher for boys (*M* = 3.5, *SD* = 2.7) compared to girls (*M* = 2.8, *SD* = 2.2).

## Discussion

This study is the first to provide psychometric evaluations and reference values for several PROMIS parent-proxy measures for children aged 5-7 years in a general population outside the U.S. Overall, PROMIS Global Health (v3.0), Anxiety (v3.0), Depressive Symptoms (v3.0), Anger (v3.0), Peer Relationships (v3.0), Mobility (v3.0), Fatigue (v3.0), Sleep Disturbance (v1.0) and Pain Intensity (v1.0) measures demonstrated acceptable psychometric properties in the Dutch general population. Structural validity analyses showed that IRT-assumptions were met across all measures, supported by good CFA fit indices and strong exploratory bi-factor indices. Monotonicity was confirmed by acceptable Hi and H coefficients. Nevertheless, IIO results were low across most domains (HT = 0.04–0.36). Since IIO is not a required assumption for GRMs, these results were considered descriptive and do not affect the conclusion regarding structural validity. The lower HT-values likely reflect the limited number of respondents at the extreme ends of the latent trait distribution, which is often seen in general-population samples. Item misfit was identified for five items. One item from the PROMIS Global Health scale (*PedGlobal2_PXR1*) showed misfit, probably due to its low discrimination parameter, weak loading on the general factor, and poor monotonicity. This might be explained by the universal nature of sadness, which is not necessarily related to a child’s overall health status. This suggests that the PROMIS Global Health scale may not fully reflect a single construct, but rather combines multiple domains (e.g., physical, emotional, and social health), which complicates the application of unidimensional CFA and IRT models. One item from the PROMIS Fatigue measure (*Pf4fatigue11r*) also showed misfit, although no clear explanation was found. Three items from the PROMIS Sleep Disturbance measure showed misfit: two reversed items (*sq007p_r* and *sq020p_r*), which might have introduced response difficulties, and one item (*sq023p*), which contains the more subjective phrase *“too early”* compared to the more specific phrase *“at night”* used in other items. Nevertheless, the impact of item misfit was considered negligible for these five items based on item information and item-fit plots. Construct validity analyses provided evidence that PROMIS scores assess similar constructs as corresponding PedsQL (sub)scales. Across all reliability analyses (SE(*θ*) ≤0.32, TIFs, and marginal reliability coefficients), scores showed good to excellent reliability for all measures except Mobility, with reliability exceeding 0.90 at the sample mean and extending into clinically relevant ranges. Mobility showed lower reliability at the sample mean, but adequate precision in the lower to middle *θ*-range. PROMIS CAT was most efficient, providing more information per item than the item bank, short form, or PedsQL measures. Regarding measurement invariance, one item from the PROMIS Depressive Symptoms measure (*Pf2depr1r2*) and two items from the Mobility measure (*Pf3mobil8r2* and *Pf4mobil9r2*) were flagged for DIF. However, the impact on the total scores was considered negligible, based on TCCs and differences in T-scores and reliability estimates between the original application forms and those without item(s) flagged for DIF.

The adequate psychometric properties of PROMIS Anxiety [20, 43, 44, 52–56], Depressive Symptoms [20, 43, 44, 52–56], Anger [27, 43, 44, 52, 54, 55], Peer Relationships [21, 43, 44, 52, 54–56], and Fatigue [43–45, 52, 54, 55] measures align with studies conducted in U.S. and Dutch general populations, although these studies did not specifically focus on children aged 5-7 years. Results for the PROMIS Global Health scale were also comparable to other studies in the Dutch general population, except for the higher fit indices observed in our exploratory bi-factor analysis [22, 47]. Regarding structural validity evidence for scores on the PROMIS Sleep Disturbance measure, our findings were consistent with those of the U.S. general population [57], but showed better fit indices than reported in Dutch pediatric and adult general populations [26, 58–60]. Due to differences in the types of measures used –parent-proxy, pediatric, and adult– comparisons should be interpreted with caution. In contrast, the PROMIS Mobility scores showed notably lower reliability compared to prior studies [43, 44, 52, 54, 55, 61], which might be explained by the high percentage of participants with extreme raw summed scores in this study (i.e., those with the best possible functioning). The skewed distribution of PROMIS Mobility scores aligns with expectations, as our study sample consisted only of parents from the general population, whereas the U.S. sample included parents from both general and clinical populations [52]. In accordance with this explanation, TIFs and the comparison between theoretical and empirical marginal reliability coefficients showed that PROMIS Mobility provided adequate measurement precision in the lower to middle range of *θ*, but limited precision at higher *θ*-levels. This supports the interpretation that the lower reliability estimates observed in our study reflect the sample characteristics rather than limitations of the measure itself.

Reference values for PROMIS v1.0/v3.0 measures obtained in this study (*M* =[44.8–54.1]; *SD* =[7.4–11.8]) suggest worse outcomes in the Dutch general population compared to U.S. reference values across most constructs, except for Peer Relationships. This discrepancy may partly reflect age differences in the samples, as the U.S. data included children aged 8-17 years, while this study focused exclusively on children aged 5-7 years [52]. Research has shown that the prevalence of certain health outcomes varies with age; for example, back pain and depressive symptoms are more common in older children, while asthma and abdominal pain are more frequent in younger children [62, 63]. These age-related differences can affect how symptoms are experienced and reported. However, cross-country differences should be interpreted with caution, as no cross-cultural language DIF was examined. Therefore, observed differences may partly reflect translation or cultural factors rather than population differences. Reference values for PROMIS v2.0 measures (*M* =[44.5–50.7]; *SD* = [9.1–12.5]) differed from those of v3.0, although identical response patterns were used to calculate T-scores. This difference is explained by the use of different IRT item parameters in scoring. The T-score differences between versions illustrate that the PROMIS version used should be taken into account and reported when interpreting and comparing results. Importantly, when comparing the mean SE(*θ*) between v3.0 and v2.0, v3.0 generally demonstrated lower mean SE(*θ*), indicating higher score precision. This improvement in measurement precision has positive implications for the use of CAT, as fewer items could be needed to obtain reliable T-scores when using v3.0, thereby possibly reducing the response burden and increasing efficiency in clinical or research settings.

A known limitation of parent-proxy measures is that parents may find it difficult to observe and evaluate their child’s physical, mental, and social health [64]. This could partly explain the observed item misfit in this study, particularly for the reversed items, which introduce additional response difficulties [65]. Therefore, the Dutch reference values presented in this study should not be generalized to child self-reports, as parent- and child-reported outcomes may differ.

Strengths of this study include the large sample size for psychometric evaluation and a reference sample closely aligned with the Dutch population on key demographic characteristics, which strengthened the accuracy of the derived reference values. Nevertheless, this study has some limitations as well. Firstly, the influence of language differences between U.S. and Dutch populations was not examined, as U.S. item parameters were unavailable at the time of analyses. Secondly, participants were recruited through an internet survey provider, which may have introduced selection bias by attracting respondents who are more familiar with technology, and have higher socioeconomic status. Although the purposive sampling method ensured alignment with the Dutch general population on selected demographic characteristics, the 13.0% response rate means that non-response bias cannot be ruled out. No information was available for non-respondents, as they did not provide informed consent, and thus non-response analyses could not be performed. Thirdly, the survey was programmed with forced completion to avoid missing data. While this eliminated the need for data imputation, it might have increased response burden and the risk of satisficing, thereby potentially reducing response quality.

Overall, we consider PROMIS parent-proxy Global Health, Anxiety, Depressive Symptoms, Anger, Peer Relationships, Mobility, Fatigue, Sleep Disturbance, and Pain Intensity measures suitable for use in both research and clinical practice. These measures are available through the Dutch-Flemish PNC. T-scores should be calculated using the HealthMeasures Scoring Service or the U.S. Scoring Manuals (based on the item parameters used in the U.S.), following international recommendations [29]. These T-scores can then be compared to the Dutch general population using reference values established in this study to facilitate interpretation of scores. However, comparisons with U.S. reference values should be interpreted with caution, as cross-cultural language differences were not examined. We recommend using PROMIS v3.0, where available, to align with international standards. The current study contributes to growing evidence supporting the use of PROMIS both in the Netherlands and globally by demonstrating its psychometric properties and providing Dutch reference values for children aged 5–7 years. Future research should examine language-related DIF between U.S. and Dutch samples to strengthen cross-cultural validity and address potential differences in item interpretation.

## Conclusion

Acceptable psychometric properties of scores were found for Dutch-Flemish versions of PROMIS parent-proxy Anxiety (v3.0), Depressive Symptoms (v3.0), Peer Relationships (v3.0), Mobility (v3.0), Fatigue (v3.0), Sleep Disturbance (v1.0) item banks, as well as for the Global Health scale (v3.0), Anger short form 5a (v3.0), and Pain Intensity item (v1.0). Reliability was high for all measures except Mobility, which showed limited precision at higher functioning levels. This study also provides reference values for the Dutch general population of children aged 5-7 years for both v1.0/v3.0 and v2.0, which can be used in clinical and research settings.

## Electronic supplementary material

Below is the link to the electronic supplementary material.

Supplementary Material 1

## Data Availability

The datasets used and/or analyzed during the current study are available from the corresponding author on reasonable request. Supplementary information can be accessed in the online version at [xxxx].

## Abbreviations

PRO: Patient-Reported Outcome
PROM: Patient-Reported Outcome Measure
PROMIS®: Patient-Reported Outcomes Measurement Information System
CAT: Computerized Adaptive Testing
IRT: Item-Response Theory
PNC: PROMIS National Center
GRM: Graded Response Model
PedsQL: Pediatric Quality of Life Inventory
GCS: Generic Core Scales
MFS: Multidimensional Fatigue Scale
DIF: Differential Item Functioning
SE: Standard Error
EAP: Expected A Posteriori
MPWI: Maximum Posterior Weighted Information
SD: Standard Deviation
NA: Not Applicable
CFA: Confirmatory Factor Analysis
RMSEA: Root Mean Square Error of Approximation
ECV: Explained Common Variance
CFI: Comparative Fit Index
TLI: Tucker-Lewis Index
SRMR: Standardized Root Mean Residual
UD: Uniform Differential Item Functioning
NUD: Non-Uniform Differential Item Functioning
TCC: Test Characteristic Curve
EF: Emotional Functioning
SF: Social Functioning
PF: Physical Functioning
