# Supplementary material for: Psychometric properties and reference values of nine PROMIS parent-proxy measures for children aged 5 to 7 years in the Netherlands
Source: J Patient Rep Outcomes. 2026 Mar 2;10:52. doi: 10.1186/s41687-026-01027-y (PMC13057081; doi:10.1186/s41687-026-01027-y)
Supplement: Supplementary file 1 — Supplementary Material 1 [file 41687_2026_1027_MOESM1_ESM.docx]

**Appendix A**

**Table A1**. Differences between PROMIS parent-proxy version 2.0 and version 3.0 measures

| **Version 2.0** | | **Version 3.0** | |
| --- | --- | --- | --- |
| **Item ID** | **Item categories** | **Item ID** | **Item categories** |
| **Anxiety** | | | |
| Pf2anxiety3r | 1,2,3,4,4 | Pf2anxiety3r2 | 1,2,3,4,5 |
| **Depressive Symptoms** | | | |
| Pf2depr1r2 | 1,2,3,4,5 | Pf2depr1r2 | 1,2,3,4,4 |
| **Peer Relationships** | | | |
| Pf4socabil15r | 5,4,3,2,1 | Removed |  |
| **Mobility** | | | |
| Pf3mobil2r2 | 5,4,3,2,1 | Pf3mobil2r2 | 4,3,2,1,1 |
| Pf3mobil12r2 | 5,4,3,2,1 | Pf3mobil12r2 | 4,3,2,1,1 |
| Pf4mobil10r2 | 5,4,3,2,1 | Pf4mobil10r2 | 4,3,2,1,1 |
| Pf4mobil2r2 | 5,4,3,2,1 | Pf4mobil2r2 | 4,3,2,1,1 |
| Pf3mobil9r2 | 5,4,3,2,1 | Pf3mobil9r2 | 4,3,2,1,1 |
| Pf3mobil3r2 | 5,4,3,2,1 | Pf3mobil3r2 | 4,3,2,1,1 |
| Pf2mobil4r2 | 5,4,3,2,1 | Pf2mobil4r2 | 4,3,2,1,1 |
| Pf4mobil3r2 | 5,4,3,2,1 | Pf4mobil3r2 | 4,3,2,1,1 |
| Pf2mobil7r2 | 5,4,3,2,1 | Pf2mobil7r2 | 4,3,2,1,1 |
| Pf2mobil6r2 | 5,4,3,2,1 | Pf2mobil6r2 | 4,3,2,1,1 |
| Pf1mobil5r2 | 5,4,3,2,1 | Pf1mobil5r2 | 4,3,2,1,1 |
| Pf3mobil8r2 | 5,4,3,2,1 | Pf3mobil8r2 | 4,3,2,1,1 |
| Pf4mobil6r2 | 5,4,3,2,1 | Pf4mobil6r2 | 4,3,2,1,1 |
| Pf4mobil4r2 | 5,4,3,2,1 | Pf4mobil4r2 | 4,3,2,1,1 |
| Pf1mobil6r2 | 5,4,3,2,1 | Pf1mobil6r2 | 4,3,2,1,1 |
| Pf4mobil9r2 | 5,4,3,2,1 | Pf4mobil9r2 | 4,3,2,1,1 |
| Pf1mobil1r2 | 5,4,3,2,1 | Pf1mobil1r2 | 4,3,2,1,1 |
| Pf3mobil10r2 | 5,4,3,2,1 | Pf3mobil10r2 | 4,3,2,1,1 |
| Pf1mobil3r | 5,4,3,2,1 | Removed |  |
| Pf4mobil7r2 | 5,4,3,2,1 | Removed |  |
| Pf3mobil5r2 | 5,4,3,2,1 | Removed |  |
| **Fatigue** | | | |
| Pf4fatigue4r | 1,2,3,4,5 | Pf4fatigue4r2 | 1,2,3,4,4 |
| Pf4fatigue12r2 | 1,2,3,4,5 | Pf4fatigue12r2 | 1,2,3,4,4 |
| Pf4fatigue5r2 | 1,2,3,4,4 | Pf4fatigue5r2 | 1,2,3,4,5 |
| Pf1fatigue3r2 | 1,2,3,4,4 | Pf1fatigue3r2 | 1,2,3,4,5 |

**Appendix B**

**Table B1.** Results regarding GRM-model fit, parameters, and Differential Item Functioning for each PROMIS parent-proxy measure (*n*=529)

| PROMIS parent-proxy item | GRM-model fit | GRM-model item parameters | | | | | Differential Item Functioning | | | | | | | | | | | | | |
| --- | --- | --- | --- | --- | --- | --- | --- | --- | --- | --- | --- | --- | --- | --- | --- | --- | --- | --- | --- | --- |
|  | **S-X^2^**  ***p*-value** | **a** | **b1** | **b2** | **b3** | **b4** | **Age**  **parent** | | **Sex**  **parent** | | **Education parent** | | **Age**  **child** | | **Sex**  **child** | | **Ethnicity**  **child** | | | |
|  |  |  |  |  |  |  | **R^2^** | **DIF** | **R^2^** | **DIF** | **R^2^**  **mean** | **DIF** | **R^2^**  **mean** | **DIF** | **R^2^** | **DIF** | **R^2^**  **mean** | | **DIF** | |
| Global Health - 7 (v3.0) | | | | | | | | | | | | | | | | | | | | |
| Global01_PXR1 | 0.111 | 4.199 | -2.558 | -1.634 | -0.541 | 0.566 | 0.000 |  | 0.001 |  | 0.001 |  | 0.002 |  | 0.001 |  | 0.002 |  | | |
| Global02_PXR1 | 0.003 | 4.234 | -2.551 | -1.755 | -0.800 | 0.440 | 0.000 |  | 0.001 |  | 0.003 |  | 0.001 |  | 0.000 |  | 0.001 |  | | |
| Global03_PXR1 | 0.112 | 4.992 | -2.485 | -1.498 | -0.648 | 0.393 | 0.000 |  | 0.000 |  | 0.003 |  | 0.000 |  | 0.001 |  | 0.003 |  | | |
| Global04_PXR1 | 0.021 | 3.001 | -2.894 | -1.703 | -0.685 | 0.513 | 0.000 |  | 0.001 |  | 0.003 |  | 0.000 |  | 0.000 |  | 0.001 |  | | |
| PedGlobal2_PXR1 | 0.001 | 1.013 | -6.680 | -3.115 | -0.438 | 3.030 | 0.002 |  | 0.000 |  | 0.001 |  | 0.002 |  | 0.000 |  | 0.002 |  | | |
| PedGlobal5_PXR1 | 0.078 | 1.189 | -4.934 | -3.219 | -2.054 | 1.087 | 0.007 |  | 0.013 |  | 0.005 |  | 0.001 |  | 0.000 |  | 0.009 |  | | |
| PedGlobal6_PXR1 | 0.012 | 1.091 | -5.289 | -3.741 | -2.159 | 1.474 | 0.001 |  | 0.008 |  | 0.002 |  | 0.007 |  | 0.000 |  | 0.003 |  | | |
| Anxiety item bank (v3.0) | | | | | | | | | | | | | | | | | | | | |
| Pf1anxiety8r ^a^ | 0.078 | 1.828 | -1.074 | 0.300 | 2.077 | 3.347 | 0.001 |  | 0.002 |  | 0.004 |  | 0.002 |  | 0.001 |  | 0.002 |  | | |
| Pf2anxiety2r ^a^ | 0.541 | 2.664 | -0.744 | 0.492 | 1.801 | 2.668 | 0.000 |  | 0.006 |  | 0.008 |  | 0.001 |  | 0.000 |  | 0.001 |  | | |
| Pf2anxiety9r ^a^ | 0.041 | 2.903 | -0.664 | 0.480 | 1.817 | 2.697 | 0.000 |  | 0.002 |  | 0.002 |  | 0.003 |  | 0.001 |  | 0.001 |  | | |
| Pf2anxiety1r ^a^ | 0.665 | 3.096 | 0.206 | 1.034 | 2.097 | 2.729 | 0.001 |  | 0.001 |  | 0.002 |  | 0.003 |  | 0.002 |  | 0.002 |  | | |
| Pf2anxiety5r ^a^ | 0.835 | 3.574 | 0.310 | 1.158 | 1.921 | 2.642 | 0.000 |  | 0.001 |  | 0.002 |  | 0.003 |  | 0.003 |  | 0.002 |  | | |
| Pf1anxiety1r ^a^ | 0.573 | 3.439 | 0.227 | 1.040 | 1.827 | 2.475 | 0.002 |  | 0.000 |  | 0.001 |  | 0.000 |  | 0.000 |  | 0.002 |  | | |
| Pf1anxiety3r ^a^ | 0.063 | 3.700 | 0.269 | 1.129 | 1.834 | 2.662 | 0.002 |  | 0.000 |  | 0.002 |  | 0.003 |  | 0.001 |  | 0.004 |  | | |
| Pf2anxiety4r ^a^ | 0.137 | 2.909 | 0.124 | 0.952 | 1.779 | 2.885 | 0.002 |  | 0.002 |  | 0.002 |  | 0.002 |  | 0.000 |  | 0.004 |  | | |
| Pf1anxiety6r | 0.172 | 3.307 | 0.371 | 1.123 | 1.834 | 2.466 | 0.000 |  | 0.001 |  | 0.003 |  | 0.006 |  | 0.000 |  | 0.003 |  | | |
| Pf1anxiety5r | 0.054 | 2.314 | 0.269 | 1.167 | 2.113 | 2.992 | 0.000 |  | 0.000 |  | 0.007 |  | 0.004 |  | 0.000 |  | 0.004 |  | | |
| Pf2anxiety3r | 0.082 | 3.091 | 0.693 | 1.378 | 2.061 | 2.831 | 0.004 |  | 0.003 |  | 0.007 |  | 0.003 |  | 0.001 |  | 0.005 |  | | |
| Pf1anxiety9r | 0.014 | 3.017 | 0.340 | 1.124 | 1.942 | 2.683 | 0.000 |  | 0.003 |  | 0.000 |  | 0.002 |  | 0.002 |  | 0.010 |  | | |
| Pf2anxiety7r | 0.224 | 3.750 | 0.724 | 1.299 | 1.856 | 2.617 | 0.001 |  | 0.001 |  | 0.005 |  | 0.003 |  | 0.000 |  | 0.003 |  | | |
| Depressive symptoms item bank (v3.0) | | | | | | | | | | | | | | | | | | | |  |
| Pf2depr7r ^a^ | 0.071 | 2.527 | 0.144 | 1.136 | 2.134 | 2.942 | 0.000 |  | 0.007 |  | 0.003 |  | 0.001 |  | 0.000 |  | 0.001 |  | | |
| Pf1depr7r ^a^ | 0.157 | 3.972 | 0.642 | 1.342 | 2.000 | 2.719 | 0.001 |  | 0.000 |  | 0.003 |  | 0.001 |  | 0.000 |  | 0.007 |  | | |
| Pf1depr5r ^a^ | 0.081 | 2.752 | 0.400 | 1.157 | 1.983 | 2.902 | 0.001 |  | 0.003 |  | 0.003 |  | 0.005 |  | 0.000 |  | 0.002 |  | | |
| Pf2depr10r ^a^ | 0.448 | 3.814 | 0.440 | 1.297 | 1.908 | 2.462 | 0.000 |  | 0.000 |  | 0.002 |  | 0.001 |  | 0.004 |  | 0.002 |  | | |
| Pf2depr3r ^a^ | 0.004 | 2.247 | -0.618 | 0.578 | 2.094 | 2.958 | 0.003 |  | 0.010 |  | 0.003 |  | 0.001 |  | 0.000 |  | 0.002 |  | | |
| Pf2depr11r | 0.416 | 3.776 | 0.345 | 1.188 | 1.973 | 2.898 | 0.000 |  | 0.000 |  | 0.005 |  | 0.003 |  | 0.000 |  | 0.005 |  | | |
| Pf1depr8r | 0.379 | 4.495 | 0.652 | 1.361 | 1.914 | 2.457 | 0.000 |  | 0.000 |  | 0.010 |  | 0.004 |  | 0.000 |  | 0.007 |  | | |
| Pf2depr6r ^a^ | 0.128 | 4.623 | 0.651 | 1.364 | 2.035 | 2.650 | 0.002 |  | 0.003 |  | 0.000 |  | 0.003 |  | 0.000 |  | 0.004 |  | | |
| Pf1depr4r | 0.597 | 3.598 | 0.519 | 1.306 | 2.044 | 3.177 | 0.001 |  | 0.000 |  | 0.003 |  | 0.005 |  | 0.001 |  | 0.011 |  | | |
| Pf2depr1r2 | 0.072 | 3.998 | 0.755 | 1.371 | 2.004 | - | 0.001 |  | 0.011 |  | 0.018 | UD & NUD | 0.006 |  | 0.002 |  | 0.002 |  | | |
| Pf2depr8r | 0.011 | 3.031 | 0.478 | 1.180 | 1.970 | 2.944 | 0.001 |  | 0.004 |  | 0.002 |  | 0.001 |  | 0.001 |  | 0.005 |  | | |
| Pf2depr2r | 0.063 | 3.891 | 0.658 | 1.329 | 2.020 | 2.761 | 0.001 |  | 0.002 |  | 0.004 |  | 0.002 |  | 0.002 |  | 0.002 |  | | |
| Pf1depr1r | 0.296 | 1.879 | 0.296 | 1.286 | 2.391 | 4.276 | 0.002 |  | 0.000 |  | 0.005 |  | 0.002 |  | 0.000 |  | 0.000 |  | | |
| Anger short form 5a (v3.0) | | | | | | | | | | | | | | | | | | | |  |
| Pf1anger1r | 0.002 | 2.517 | -1.377 | -0.210 | 1.718 | 3.298 | 0.000 |  | 0.014 |  | 0.001 |  | 0.002 |  | 0.000 |  | 0.004 |  | | |
| Pf1anger5r | 0.030 | 3.840 | -0.474 | 0.283 | 1.520 | 2.671 | 0.000 |  | 0.000 |  | 0.001 |  | 0.002 |  | 0.001 |  | 0.003 |  | | |
| Pf1anger3r | 0.014 | 3.512 | -0.063 | 0.689 | 1.646 | 2.999 | 0.001 |  | 0.004 |  | 0.003 |  | 0.001 |  | 0.000 |  | 0.000 |  | | |
| Pf1anger10r | 0.281 | 2.758 | -0.620 | 0.364 | 1.755 | 3.032 | 0.002 |  | 0.004 |  | 0.003 |  | 0.001 |  | 0.000 |  | 0.001 |  | | |
| Pf1anger8r | 0.057 | 2.644 | 0.037 | 0.971 | 1.929 | 2.784 | 0.003 |  | 0.011 |  | 0.002 |  | 0.000 |  | 0.005 |  | 0.004 |  | | |
| Peer relationships item bank (v3.0) | | | | | | | | | | | | | | | | | | | |  |
| Pf3socrole4r ^a^ | 0.071 | 2.315 | -2.733 | -2.255 | -0.854 | 0.931 | 0.003 |  | 0.004 |  | 0.000 |  | 0.001 |  | 0.001 |  | 0.002 |  | | |
| Pf1socabil6r | 0.005 | 3.714 | -2.578 | -1.764 | -0.977 | 0.445 | 0.002 |  | 0.003 |  | 0.002 |  | 0.002 |  | 0.000 |  | 0.003 |  | | |
| Pf1socrole2r | 0.101 | 3.324 | -2.525 | -2.121 | -1.251 | 0.178 | 0.000 |  | 0.000 |  | 0.003 |  | 0.003 |  | 0.005 |  | 0.003 |  | | |
| Pf2socabil9r ^a^ | 0.041 | 3.018 | -2.829 | -2.173 | -1.189 | 0.384 | 0.000 |  | 0.002 |  | 0.003 |  | 0.001 |  | 0.000 |  | 0.000 |  | | |
| Pf3socabil4r ^a^ | 0.032 | 3.253 | -2.579 | -1.686 | -0.867 | 0.309 | 0.001 |  | 0.001 |  | 0.003 |  | 0.004 |  | 0.000 |  | 0.003 |  | | |
| Pf4socabil12r ^a^ | 0.504 | 3.381 | -2.559 | -1.901 | -0.738 | 0.723 | 0.000 |  | 0.000 |  | 0.000 |  | 0.002 |  | 0.000 |  | 0.001 |  | | |
| Pf3socabil9r ^a^ | 0.752 | 3.697 | -2.717 | -1.949 | -1.013 | 0.355 | 0.000 |  | 0.003 |  | 0.004 |  | 0.003 |  | 0.001 |  | 0.008 |  | | |
| Pf2socrole4r ^a^ | 0.259 | 4.171 | -2.678 | -1.947 | -0.870 | 0.440 | 0.000 |  | 0.001 |  | 0.002 |  | 0.001 |  | 0.002 |  | 0.001 |  | | |
| Pf1socabil2r ^a^ | 0.346 | 3.779 | -2.431 | -1.847 | -0.822 | 0.550 | 0.000 |  | 0.000 |  | 0.005 |  | 0.004 |  | 0.004 |  | 0.001 |  | | |
| Pf4socabil4r | 0.347 | 3.816 | -2.518 | -1.828 | -0.963 | 0.342 | 0.002 |  | 0.001 |  | 0.002 |  | 0.003 |  | 0.000 |  | 0.000 |  | | |
| Pf4socabil10r | 0.001 | 2.785 | -2.703 | -1.846 | -0.681 | 0.668 | 0.002 |  | 0.000 |  | 0.001 |  | 0.004 |  | 0.002 |  | 0.003 |  | | |
| Pf2socabil7r | 0.083 | 2.964 | -2.855 | -2.107 | -1.036 | 0.480 | 0.000 |  | 0.000 |  | 0.001 |  | 0.002 |  | 0.003 |  | 0.001 |  | | |
| Pf4socrole3r | 0.032 | 3.275 | -2.801 | -1.874 | -0.853 | 0.619 | 0.002 |  | 0.000 |  | 0.003 |  | 0.001 |  | 0.000 |  | 0.005 |  | | |
| Pf2socabil6r | 0.052 | 2.394 | -3.394 | -2.025 | -0.851 | 0.605 | 0.001 |  | 0.000 |  | 0.002 |  | 0.004 |  | 0.001 |  | 0.002 |  | | |
| Mobility item bank (v3.0) | | | | | | | | | | | | | | | | | | | |  |
| Pf3mobil2r2 | 0.324 | 3.346 | -2.077 | -1.528 | -1.100 | - | 0.002 |  | 0.002 |  | 0.000 |  | 0.005 |  | 0.000 |  | 0.001 |  | | |
| Pf3mobil12r2 | 0.323 | 7.459 | -2.075 | -1.625 | -1.347 | - | 0.000 |  | 0.002 |  | 0.002 |  | 0.009 |  | 0.000 |  | 0.002 |  | | |
| Pf4mobil10r2 | 0.248 | 6.960 | -1.910 | -1.581 | -1.264 | - | 0.001 |  | 0.000 |  | 0.001 |  | 0.013 |  | 0.001 |  | 0.010 |  | | |
| Pf4mobil2r2 | 0.025 | 7.915 | -1.944 | -1.631 | -1.158 | - | 0.001 |  | 0.001 |  | 0.002 |  | 0.008 |  | 0.005 |  | 0.000 |  | | |
| Pf3mobil9r2 ^a^ | 0.044 | 9.394 | -1.959 | -1.627 | -1.256 | - | 0.004 |  | 0.002 |  | 0.000 |  | 0.001 |  | 0.000 |  | 0.001 |  | | |
| Pf3mobil3r2 ^a^ | 0.020 | 12.623 | -1.883 | -1.667 | -1.269 | - | 0.000 |  | 0.001 |  | 0.001 |  | 0.003 |  | 0.001 |  | 0.004 |  | | |
| Pf2mobil4r2 ^a^ | 0.568 | 3.969 | -2.019 | -1.504 | -1.057 | - | 0.000 |  | 0.000 |  | 0.000 |  | 0.005 |  | 0.000 |  | 0.006 |  | | |
| Pf4mobil3r2 | 0.245 | 5.751 | -1.923 | -1.576 | -1.171 | - | 0.000 |  | 0.000 |  | 0.008 |  | 0.001 |  | 0.001 |  | 0.001 |  | | |
| Pf2mobil7r2 ^a^ | 0.176 | 4.970 | -2.059 | -1.633 | -1.197 | - | 0.001 |  | 0.000 |  | 0.000 |  | 0.001 |  | 0.002 |  | 0.001 |  | | |
| Pf2mobil6r2 | 0.541 | 6.752 | -2.011 | -1.604 | -1.239 | - | 0.006 |  | 0.002 |  | 0.000 |  | 0.007 |  | 0.001 |  | 0.001 |  | | |
| Pf1mobil5r2 | 0.280 | 4.744 | -2.090 | -1.632 | -1.093 | - | 0.006 |  | 0.001 |  | 0.000 |  | 0.002 |  | 0.001 |  | 0.003 |  | | |
| Pf3mobil8r2 ^a^ | 0.255 | 7.482 | -1.925 | -1.695 | -1.337 | - | 0.000 |  | 0.001 |  | 0.000 |  | 0.018 | UD & NUD | 0.001 |  | 0.003 |  | | |
| Pf4mobil6r2 | 0.147 | 5.585 | -1.999 | -1.658 | -1.358 | - | 0.000 |  | 0.000 |  | 0.000 |  | 0.10 |  | 0.002 |  | 0.000 |  | | |
| Pf1mobil2r | 0.342 | 2.330 | -2.535 | -1.993 | -1.405 | -0.951 | 0.000 |  | 0.003 |  | 0.000 |  | 0.011 |  | 0.001 |  | 0.001 |  | | |
| Pf4mobil4r2 ^a^ | 0.243 | 4.088 | -1.793 | -1.470 | -1.072 | - | 0.001 |  | 0.000 |  | 0.000 |  | 0.002 |  | 0.000 |  | 0.001 |  | | |
| Pf1mobil6r2 | 0.080 | 7.011 | -1.943 | -1.643 | -1.200 | - | 0.002 |  | 0.001 |  | 0.000 |  | 0.001 |  | 0.000 |  | 0.002 |  | | |
| Pf4mobil9r2 | 0.587 | 4.087 | -2.071 | -1.607 | -1.213 | - | 0.001 |  | 0.002 |  | 0.000 |  | 0.016 | UD | 0.000 |  | 0.000 |  | | |
| Pf2mobil1r | 0.193 | 0.921 | -2.918 | -2.037 | -0.917 | 0.089 | 0.000 |  | 0.003 |  | 0.000 |  | 0.001 |  | 0.000 |  | 0.004 |  | | |
| Pf1mobil1r2 ^a^ | 0.123 | 3.747 | -2.199 | -1.593 | -1.178 | - | 0.000 |  | 0.004 |  | 0.000 |  | 0.008 |  | 0.001 |  | 0.001 |  | | |
| Pf3mobil10r2 | 0.312 | 5.847 | -2.087 | -1.725 | -1.360 | - | 0.002 |  | 0.000 |  | 0.008 |  | 0.002 |  | 0.002 |  | 0.000 |  | | |
| Fatigue item bank (v3.0) | | | | | | | | | | | | | | | | | | | |  |
| Pf4fatigue4r2 ^a^ | 0.709 | 3.300 | -0.056 | 1.000 | 1.941 | - | 0.000 |  | 0.001 |  | 0.003 |  | 0.001 |  | 0.003 |  | 0.003 |  | | |
| Pf4fatigue12r2 ^a^ | 0.463 | 3.786 | 0.174 | 1.072 | 1.894 | - | 0.000 |  | 0.000 |  | 0.001 |  | 0.000 |  | 0.000 |  | 0.003 |  | | |
| Pf3fatigue7r ^a^ | 0.234 | 4.010 | 0.158 | 1.057 | 1.803 | 2.291 | 0.000 |  | 0.000 |  | 0.002 |  | 0.001 |  | 0.000 |  | 0.002 |  | | |
| Pf4fatigue8r ^a^ | 0.303 | 3.788 | 0.408 | 1.151 | 1.822 | 2.509 | 0.001 |  | 0.001 |  | 0.001 |  | 0.005 |  | 0.001 |  | 0.002 |  | | |
| Pf3fatigue5r | 0.285 | 4.277 | 0.360 | 1.162 | 1.786 | 2.356 | 0.000 |  | 0.000 |  | 0.005 |  | 0.010 |  | 0.001 |  | 0.005 |  | | |
| Pf2fatigue8r ^a^ | 0.271 | 5.093 | 0.447 | 1.283 | 1.842 | 2.693 | 0.000 |  | 0.000 |  | 0.001 |  | 0.002 |  | 0.000 |  | 0.007 |  | | |
| Pf4fatigue3r ^a^ | 0.357 | 3.625 | 0.164 | 1.021 | 1.813 | 2.591 | 0.000 |  | 0.003 |  | 0.002 |  | 0.006 |  | 0.000 |  | 0.002 |  | | |
| Pf3fatigue8r ^a^ | 0.052 | 4.805 | 0.375 | 1.232 | 1.843 | 2.403 | 0.001 |  | 0.000 |  | 0.004 |  | 0.001 |  | 0.000 |  | 0.003 |  | | |
| Pf3fatigue12r ^a^ | 0.035 | 4.771 | 0.374 | 1.171 | 1.898 | 2.575 | 0.000 |  | 0.000 |  | 0.006 |  | 0.004 |  | 0.002 |  | 0.001 |  | | |
| Pf2fatigue4r ^a^ | 0.495 | 5.440 | 0.356 | 1.123 | 1.786 | 2.507 | 0.001 |  | 0.001 |  | 0.004 |  | 0.002 |  | 0.002 |  | 0.001 |  | | |
| Pf3fatigue1r | 0.004 | 2.710 | -0.284 | 0.651 | 1.844 | 2.987 | 0.000 |  | 0.002 |  | 0.001 |  | 0.003 |  | 0.000 |  | 0.002 |  | | |
| Pf4fatigue5r2 | 0.047 | 3.106 | 0.681 | 1.454 | 2.041 | 2.964 | 0.000 |  | 0.000 |  | 0.007 |  | 0.004 |  | 0.003 |  | 0.007 |  | | |
| Pf4fatigue1r | 0.193 | 3.832 | 0.286 | 1.043 | 1.822 | 2.379 | 0.000 |  | 0.001 |  | 0.004 |  | 0.001 |  | 0.000 |  | 0.006 |  | | |
| Pf2fatigue7r | 0.157 | 4.834 | 0.511 | 1.222 | 1.818 | 2.425 | 0.001 |  | 0.003 |  | 0.001 |  | 0.004 |  | 0.000 |  | 0.002 |  | | |
| Pf3fatigue10r | 0.378 | 5.106 | 0.538 | 1.277 | 1.957 | 2.399 | 0.004 |  | 0.000 |  | 0.009 |  | 0.006 |  | 0.000 |  | 0.000 |  | | |
| Pf3fatigue4r ^a^ | 0.010 | 3.909 | 0.356 | 1.259 | 1.969 | 2.724 | 0.000 |  | 0.001 |  | 0.001 |  | 0.001 |  | 0.000 |  | 0.002 |  | | |
| Pf3fatigue11r | 0.035 | 2.915 | 0.380 | 1.196 | 1.982 | 2.686 | 0.000 |  | 0.003 |  | 0.006 |  | 0.003 |  | 0.003 |  | 0.007 |  | | |
| Pf4fatigue10r | 0.121 | 4.065 | 0.656 | 1.294 | 1.947 | 2.580 | 0.000 |  | 0.000 |  | 0.003 |  | 0.004 |  | 0.000 |  | 0.007 |  | | |
| Pf1fatigue3r | 0.020 | 3.249 | 0.586 | 1.325 | 1.937 | 2.641 | 0.000 |  | 0.000 |  | 0.002 |  | 0.005 |  | 0.000 |  | 0.002 |  | | |
| Pf1fatigue5r | 0.008 | 2.722 | 0.688 | 1.307 | 2.008 | 2.518 | 0.002 |  | 0.000 |  | 0.003 |  | 0.005 |  | 0.004 |  | 0.010 |  | | |
| Pf1fatigue1r | 0.667 | 3.765 | 0.542 | 1.238 | 1.829 | 2.509 | 0.000 |  | 0.000 |  | 0.002 |  | 0.004 |  | 0.001 |  | 0.001 |  | | |
| Pf4fatigue11r | 0.000* | 2.070 | 0.243 | 1.124 | 2.134 | 2.902 | 0.000 |  | 0.004 |  | 0.001 |  | 0.002 |  | 0.000 |  | 0.001 |  | | |
| Pf4fatigue6r | 0.074 | 4.139 | 0.635 | 1.252 | 1.916 | 2.861 | 0.000 |  | 0.003 |  | 0.000 |  | 0.001 |  | 0.006 |  | 0.007 |  | | |
| Sleep Disturbance item bank (v1.0) | | | | | | | | | | | | | | | | | | | | |
| sq005p ^a^ | 0.192 | 2.186 | -0.577 | 0.528 | 1.605 | 2.644 | 0.000 |  | 0.005 |  | 0.001 |  | 0.001 |  | 0.000 |  | 0.004 |  | | |
| sq007p_r | 0.000 | 1.549 | -1.047 | 0.850 | 1.650 | 2.540 | 0.002 |  | 0.000 |  | 0.001 |  | 0.001 |  | 0.000 |  | 0.006 |  | | |
| sq010p ^a^ | 0.225 | 2.160 | 0.097 | 0.996 | 1.969 | 2.826 | 0.002 |  | 0.001 |  | 0.001 |  | 0.003 |  | 0.003 |  | 0.006 |  | | |
| sq011p | 0.276 | 2.487 | 0.340 | 1.167 | 2.129 | 3.120 | 0.000 |  | 0.001 |  | 0.001 |  | 0.004 |  | 0.000 |  | 0.002 |  | | |
| sq012p | 0.060 | 1.935 | -0.021 | 0.817 | 1.866 | 3.138 | 0.001 |  | 0.000 |  | 0.002 |  | 0.001 |  | 0.000 |  | 0.003 |  | | |
| sq014p | 0.232 | 2.883 | 0.379 | 1.113 | 2.024 | 2.695 | 0.003 |  | 0.000 |  | 0.002 |  | 0.002 |  | 0.000 |  | 0.004 |  | | |
| sq017p ^a^ | 0.120 | 2.237 | -0.344 | 0.656 | 1.645 | 2.575 | 0.001 |  | 0.011 |  | 0.001 |  | 0.001 |  | 0.000 |  | 0.001 |  | | |
| sq020p_r ^a^ | 0.000 | 1.231 | -0.561 | 1.421 | 2.203 | 2.965 | 0.000 |  | 0.001 |  | 0.001 |  | 0.000 |  | 0.000 |  | 0.001 |  | | |
| sq021p | 0.285 | 3.834 | 0.499 | 1.185 | 1.756 | 2.297 | 0.000 |  | 0.001 |  | 0.001 |  | 0.001 |  | 0.001 |  | 0.003 |  | | |
| sq022p ^a^ | 0.014 | 3.712 | 0.270 | 0.948 | 1.701 | 2.406 | 0.001 |  | 0.005 |  | 0.006 |  | 0.000 |  | 0.001 |  | 0.001 |  | | |
| sq023p | 0.000 | 1.334 | -0.380 | 0.692 | 2.170 | 3.228 | 0.001 |  | 0.001 |  | 0.004 |  | 0.002 |  | 0.001 |  | 0.002 |  | | |
| sq036p ^a^ | 0.233 | 2.917 | 0.205 | 1.060 | 1.795 | 2.661 | 0.002 |  | 0.006 |  | 0.001 |  | 0.001 |  | 0.002 |  | 0.002 |  | | |
| sq040p | 0.269 | 4.030 | 0.232 | 1.058 | 1.729 | 2.310 | 0.003 |  | 0.002 |  | 0.004 |  | 0.002 |  | 0.000 |  | 0.003 |  | | |
| sq041p_r ^a^ | 0.973 | 5.577 | 0.597 | 1.161 | 1.744 | 2.223 | 0.000 |  | 0.003 |  | 0.003 |  | 0.005 |  | 0.000 |  | 0.001 |  | | |
| sq042p ^a^ | 0.224 | 5.676 | 0.383 | 0.997 | 1.575 | 2.093 | 0.000 |  | 0.002 |  | 0.001 |  | 0.001 |  | 0.000 |  | 0.004 |  | | |

GRM, graded response model; UD, uniform DIF; NUD, non-uniform DIF.

For scoring these measures we recommend to use the default U.S. item parameters (through HealthMeasures Scoring Service) as per PROMIS convention.

^a^ Included in the PROMIS parent-proxy short form.

**
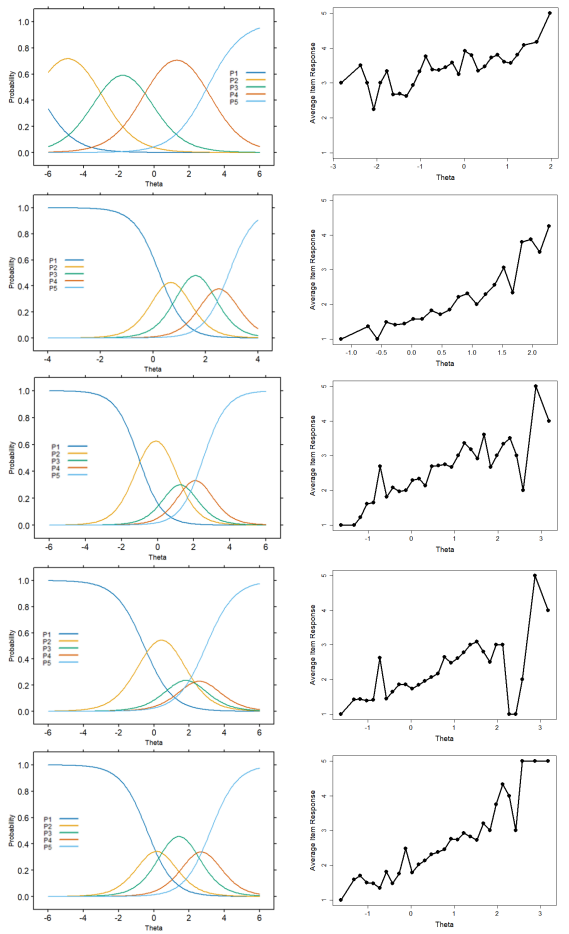
Appendix C**

**Figure C.** Average probability of selecting a response (1^st^column) and average item response (2^nd^column) across theta estimates.

First row: PROMIS parent-proxy Global Health scale item *PedGlobal2_PXR1*; Second row: PROMIS parent-proxy Fatigue measure item *Pf4fatigue11r*; Third row: PROMIS parent-proxy Sleep Disturbance measure item *sq007p_r*; Fourth row: PROMIS parent-proxy Sleep Disturbance measure item *sq020p_r*; Fifth row: PROMIS parent-proxy Sleep Disturbance measure item *sq023p*.

**Appendix D**

**
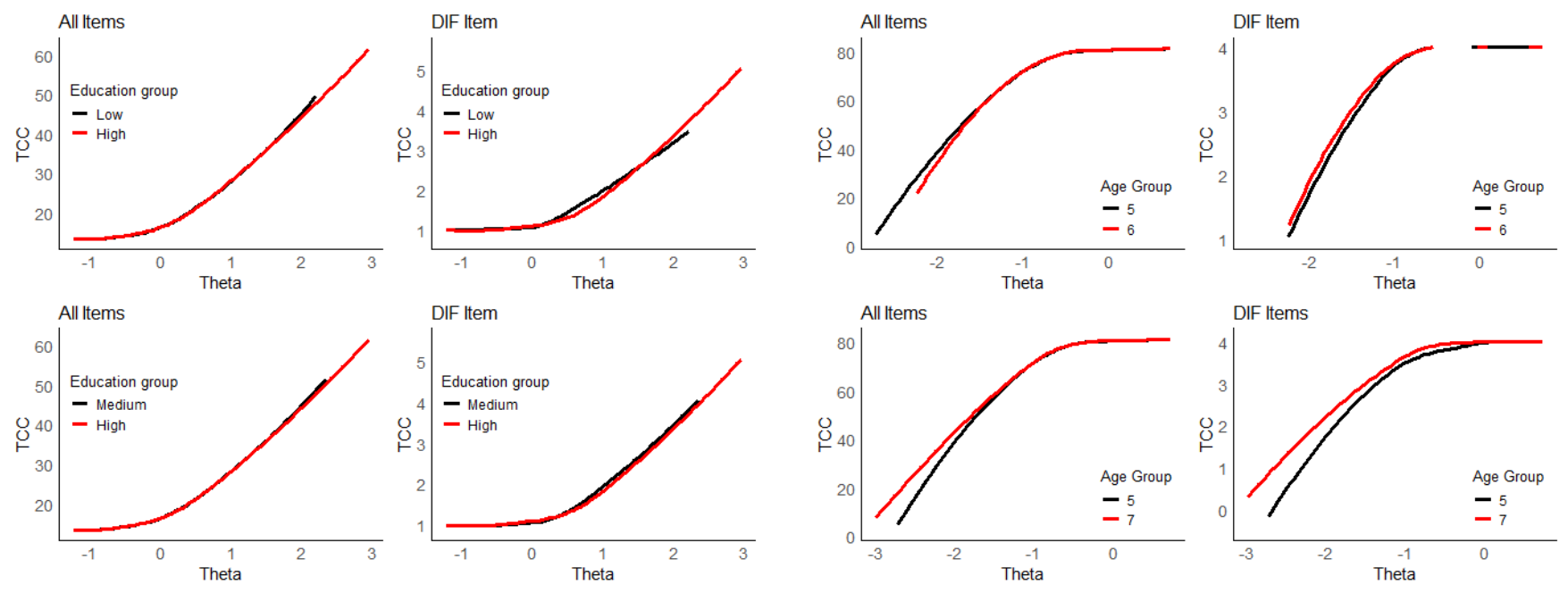

Figure D.** Test Characteristic Curves (TCCs) for the PROMIS Depressive Symptoms (columns1-2) and Fatigue (columns3-4) measures.

First and second column: PROMIS parent-proxy Depressive Symptoms measure comparing participants with low vs. high, and medium vs. high educational levels. The two plots in the first column show the total raw summed scores across all thirteen items for each educational group, while the two plots in the second column show the raw scores for only item flagged for DIF (*Pf2depr1r2*); Third and Fourth column: PROMIS parent-proxy Mobility measure comparing participants with a child aged 5 vs. 6, and 5 vs. 7. The two plots in the third column show the total raw summed scores across all twenty items for each age group, while the two plots in the fourth column show the raw scores for only item(s) flagged for DIF (*Pf3mobil8r2* and *Pf4mobil9r2*). The area between the curves within each plot illustrates the potential impact of DIF on the overall test score (*n* =529).

**Appendix E**

**
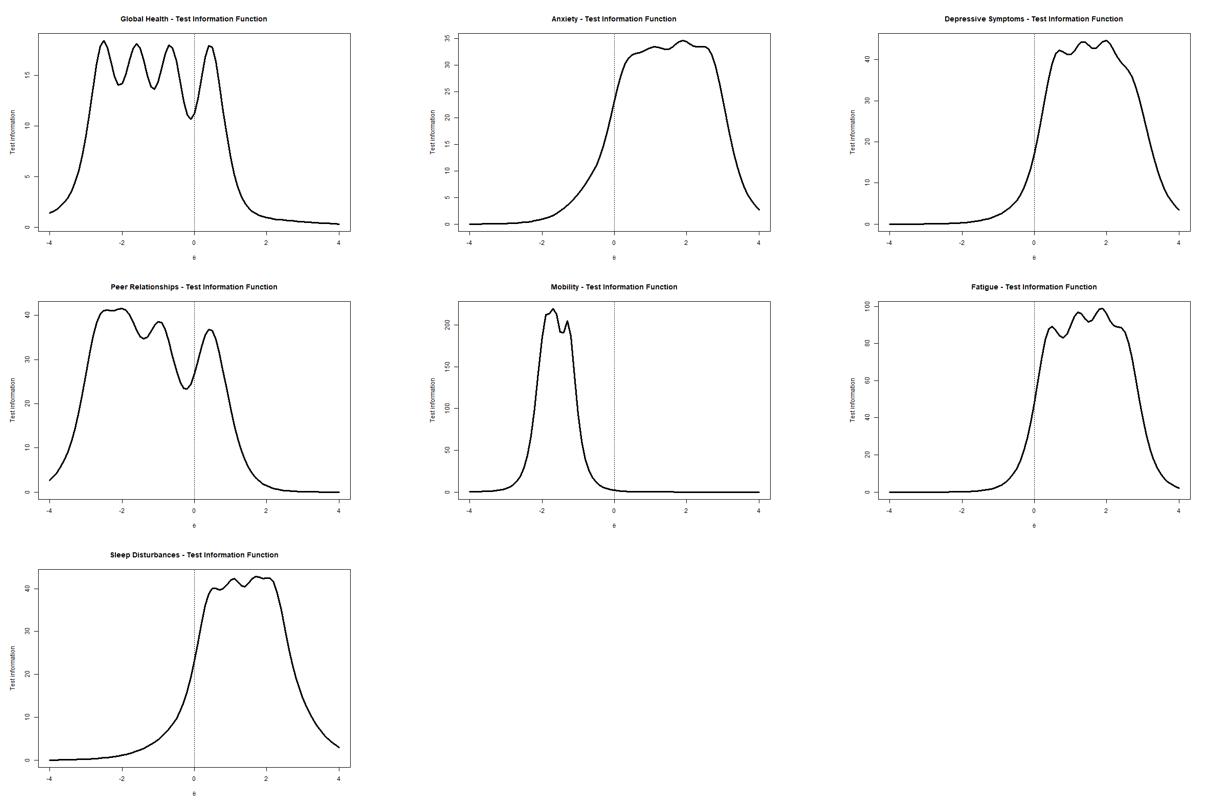

Figure E**. Test Information Curves across theta (θ) for PROMIS full item banks, using Dutch parameters (n=529).

**Table E**. Theoretical and empirical marginal reliability coefficients for PROMIS full item banks, using Dutch parameters (n=529)

| **PROMIS parent-proxy measure** | **Theoretical marginal reliability coefficient*** | **Empirical marginal reliability coefficient*** | **Difference (absolute)** |
| --- | --- | --- | --- |
| Global Health | 0.892 | 0.889 | 0.003 |
| Anxiety | 0.905 | 0.905 | 0.000 |
| Depressive Symptoms | 0.849 | 0.853 | 0.004 |
| Peer Relationships | 0.940 | 0.938 | 0.002 |
| Mobility | 0.619 | 0.563 | 0.056 |
| Fatigue | 0.874 | 0.894 | 0.020 |
| Sleep Disturbance | 0.906 | 0.903 | 0.003 |

* Marginal reliability coefficients of 0.70-0.80, 0.80-0.90, and >90 indicate acceptable, good, and excellent measurement precision respectively.
